# EEG Maturational Age Estimation: A Comparison of Visual and Automated Interpretation of the EEG in Preterm Infants

**DOI:** 10.3390/jcm14103528

**Published:** 2025-05-18

**Authors:** Elena Pavlidis, John M. O’Toole, Francesco Pisani, Geraldine B. Boylan, Nathan J. Stevenson

**Affiliations:** 1 INFANT Research Centre, University College Cork, T12 EC8P Cork, Ireland; 2 Child Neurology and Neurorehabilitation Unit, Department of Pediatrics, Regional Hospital of Bolzano (SABES-ASDAA), Teaching Hospital of Paracelsus Medical University (PMU), 39100 Bolzano, Italy; 3 Department of Paediatrics and Child Health, University College Cork, T12 YE02 Cork, Ireland; 4 Child Neuropsychiatric Unit, Department of Human Neuroscience, Sapienza University of Rome, 00185 Rome, Italy; 5 BABA Center, Department of Children’s Clinical Neurophysiology, Helsinki University Central Hospital and University of Helsinki, 09-4711 Helsinki, Finland; 6 Brain Modelling Group, QIMR Berghofer, Brisbane 4006, Australia

**Keywords:** preterm neonates, preterm newborns, conventional EEG, multichannel EEG, brain activity, maturation, algorithm, inter-rater agreement

## Abstract

**Aim:** To assess the inter-rater agreement and accuracy of human experts’ estimate of EEG maturational age (EMA) and a computer algorithm’s estimate of EMA over the first days after birth in a cohort of normally developing preterm infants. In addition, we explore the influence of post-natal age (PNA) on EMA estimates. **Methods**: Analysis was performed on EEG records from newborns determined appropriate for gestational age (GA) with favorable neurodevelopment at 2 years of age and without significant neurological compromise at time of EEG monitoring. Three 1h epochs of EEG were selected from 29 newborns with GA ranging from 23 to <32 weeks, within 72 h of birth. EEG epochs were visually assessed by two pediatric neurologists and a computer algorithm. In addition, the full, long-duration EEG recording of each newborn was assessed by one pediatric neurologist. EMA estimates were compared to GA using Pearson’s correlation coefficient (*r*) and bias and standard deviation of error (SDE). Intra-newborn agreements for the EMA estimates were assessed using standard deviation. Linear mixed-effects models were used to quantify the effect of PNA on EMA estimates. **Results**: The algorithm provides a more accurate estimate of GA using 1 h EEG epochs for correlation and bias: algorithm *r* = 0.83 vs. experts *r* = 0.60 and 0.66, *p* < 0.05 for *n* = 29; algorithm bias = −0.8 days vs. experts’ bias = 3.6 and 7.0 days, *p* < 0.01 for *n* = 29. SDE of 8.7 days for the algorithm was not significantly lower compared to the experts’ SDE = 12.4 and 13.2 days, *p* > 0.05. The algorithm has higher intra-newborn agreement compared to the experts: algorithm SDE = 4.9 days vs. experts SDE = 7.4 and 7.4 days, *p* = 0.027. For the two experts, increasing PNA is associated with an increase in EMA estimates of 6.6 days/days and 3.7 days/days. The assessment of full, long-duration EEG recordings improved the experts’ estimate of EMA (*r* = 0.82; SDE = 9.2 days). **Conclusions**: Automated analysis outperforms visual interpretation of the EEG at estimating EMA for short-duration EEG recordings. PNA is an important factor in EMA estimates.

## 1. Introduction

As the mortality rate for preterm newborns born at less than 32 weeks gestational age (GA) has decreased in recent years [1,2], an increasing number require monitoring to assess neurological function. Background EEG activity assessment in this population is extremely challenging even though it is particularly useful for prognosis. EEG features and maturational aspects in preterm newborns have been described by several authors [3,4,5,6,7,8,9,10,11,12,13,14,15]. As dysmature and disorganized EEG background activity patterns have been related to a poor prognosis [16] and EEG activity has been demonstrated to be related to structural brain development on MRI in preterm infants [17], determining whether the maturational features of preterm EEG are appropriate or not for age represents a key point in assessment.

Neurophysiologists assess brain maturation in the preterm EEG mainly by measuring the duration of burst and interburst intervals. However, the quantification of interburst intervals has proven difficult [12]. More accurate visual interpretation of the EEG should also consider amplitude, morphology, and age-specific patterns (graphoelements) and their topographical distribution. Visual interpretation of the EEG remains, nevertheless, a subjective assessment [18,19] and, for preterm EEG, requires a high degree of specialization. Recent studies overcome this subjectivity using automated algorithms to extract maturational information from the EEG [20,21,22,23,24,25,26,27,28]. These approaches could, in the future, support the assessment of preterm EEG, which remain an arduous task even for trained personnel, due to the lack of accepted and internationally used assessment schemes that identify the specific EEG features defined for preterm infants at varying ages [18,19]. Trained neurophysiologists may differentiate deviances in the maturation of the EEG of approximately two weeks. The observed changes in EEG waveforms are related to the early developmental changes in neural networks and their molecular expressions, labeled as EEG maturational age (EMA). EMA may correspond to or deviate from what is expected at a given conceptual or maturational age (MA) [28].

The aims of this study are (1) to examine the visual assessment of short- and long-duration recordings of EEG by human experts for the estimation of GA in preterm infants less than 32 weeks GA during the transitional period (within the first 72 h of birth) and (2) to compare the visual assessment to a computer algorithm. We also investigated the effect of post-neonatal age (PNA) on EMA estimates.

## 2. Materials and Methods

### 2.1. Patients

EEG data were retrospectively selected from a cohort of preterm newborns recruited between 2009 and 2011 in the Neonatal Intensive Care Unit (NICU) of Cork University Maternity Hospital, Ireland. Preterm newborns <32 weeks of gestation had continuous video and EEG monitoring over the first 3 days after birth. Data collection was approved by the Clinical Research Ethics Committee of the Cork Teaching Hospitals, Ireland. Informed and written parental consent was obtained before EEG monitoring. Data collection and all procedures in this study were conducted in accordance with the principles of the International Conference on Harmonisation of Technical Requirements for Registration of Pharmaceuticals for Human Use of Good Clinical Practice (ICH-GCP).

Newborns were included in the study if they met the following inclusion criteria:

Apgar ≥6 at 5 min after birth; absence of congenital brain malformations, chromosomal or genetic aberrations, or inborn errors of metabolism; absence of grade II or higher intra-ventricular hemorrhage (IVH), and absence of periventricular leukomalacia (PVL); absence of major comorbidities (such as sepsis, necrotizing enterocolitis, cardiac dysfunction, bronchopulmonary dysplasia) at time of EEG recording; no anti-epileptic drugs before or during EEG recording; EEG monitoring commenced within 72 h from birth; EEG normal for GA; favorable neurodevelopmental outcome at 2 years of age, or adverse outcome ascribable to later morbidities with respect to the timing of EEG recording. All the infants that did not meet these inclusion criteria were excluded.

Outcome was assessed clinically and, in the majority of infants (26/29), with the Bayley Scales of Infant Development-III at 2 years corrected age. We define favorable outcome as all three Bayley subscales (motor, cognitive, and language) above the lower limit (>85) of one standard deviation (SD) centered at the standardized mean score of 100. IVH and PVL were assessed by cranial ultrasound as part of routine clinical care within the first few days of birth. This cohort has been previously reported in O’Toole et al. 2019 [29] with one additional infant included in the present study based on EEG epoch selection.

### 2.2. EEG Data

EEG was recorded using the NicoletOne EEG monitor (Natus Medical Inc., Pleasanton, CA, USA) with 11 electrodes placed according to the international 10–20 system of electrode configuration. Electrodes covered the frontal, central, temporal, and occipital regions, with a reference electrode at Fz and a ground electrode behind the left ear. Recordings started as soon as possible after birth and when clinical staff determined the infant was stable and was continued for up to 72 h.

Three, 1h epochs, free from major or persistent artifacts, were randomly selected in the EEG of each newborn (GBB), without any further criteria of exclusion or inclusion [28].

Each EEG epoch was visually assessed using a bipolar montage with both transverse and longitudinal chains. The algorithm used a bipolar montage of F3-C3, C3-T3, Cz-C3, C3-O1, F4-C4, C4-T4, Cz-C4, C4-O2.

Two pediatric neurologists experienced in neonatal EEG evaluation (EP and FP) independently evaluated the EEG recordings to establish the EMA. They were blinded to all clinical data, including PNA, and the selection of EEG epochs. They were also unaware of which epochs belonged to the same newborn. Reviewers were not restricted to specific interpretation criteria and were free to use their own experience in determining the GA. This involved the consideration of several features, such as sleep/wake cycles, burst and interburst interval duration, amplitude, morphology, frequencies, presence, and topography of specific graphoelements. A single reviewer (EP) also evaluated the entire, long-duration EEG recording as well as the selected 1 h epochs.

### 2.3. Statistical Analysis

An automated method was also used to estimate the EMA. The algorithm was developed using quantitative EEG features combined with a machine-learning model (a support vector machine). PNA for each epoch was included with the quantitative EEG features in the machine-learning model. Details on the development of the algorithm can be found in reference [28]. The testing results of this algorithm, from a subset of the leave-one-out-cross-validation in [28], were used as the EMA estimates for this study.

We compared the EMA estimates to the recorded obstetric estimate of GA (determined from the date of the mother’s last menstrual period and confirmed by antenatal ultrasound, or in the case of in vitro fertilization, using the date of insemination). We used five measures to quantify the error of the estimates between EMA and GA, namely Pearson’s correlation coefficient, bias, standard deviation of error (SDE), and the percentage of EEG epochs correctly identified within 1 week and within 2 weeks of GA [28]. Confidence intervals (95%) were generated for all metrics using 1000 iterations of a bootstrap resampling approach.

For pair-wise comparisons among the EMA estimates, we used the William’s test for dependent correlations for the correlation coefficients, *t*-tests for error bias, and the Brown–Forsythe test for the SDE. To account for the repeated measures in the data, metrics were averaged per newborn before performing the different statistical tests. Intra-newborn agreement was assessed by calculating the SD of the EMA estimates within the 3 epochs per newborn. An omnibus test (one-way ANOVA) was used to determine differences in intra-infant agreement among the EMA estimates. If significant, a pair-wise analysis between the experts and the algorithm was calculated using a *t*-test.

Many clinical and biological factors could influence EMA estimates over the postnatal transitional period. A previous study has shown that PNA has a significant impact on the evolution of the EEG over this time frame [30]. To quantify the potential influence of PNA on the experts’ EMA estimates, we employed a linear mixed-effect model. The random effect (intercept) of the model accounts for repeated measures from each newborn. The fixed effects include PNA and GA with the EMA estimate as the predictor of the model. To determine if PNA is a significant variable in the model, and therefore associated with the EMA estimate, we compared model types using a backward selection process, as described in O’Toole et al. 2019 [29]. Simpler models are favored over more complex models unless there is a significant improvement in model fit. The log-likelihood ratio test was used to compare models with a level of significance of 0.05. Significance for the fixed effects was determined using Satterthwaite’s method, and 95% confidence intervals for fixed effects were computed using a bootstrap method with 1000 resampling iterations. Adjusted correlation values (*R*-adjusted) are also presented as a metric of model fit [30]. This whole process was repeated for each of the experts’ EMA estimates.

Statistical analysis was performed using *R* (version 3.6.1, *R* Foundation for Statistical Computing, Vienna, Austria, http://www.R-project.org/) with the *lme4* package (version 1.1-21) for the mixed-effects models. The Holm procedure was used to correct for multiple comparisons. All tests are two-sided with statistical significance defined as *p* < 0.05. The mean (SD) is used to represent vector quantities.

## 3. Results

### 3.1. Patients

Out of 80 patients, we included 29 patients that meet the inclusion criteria. The infants were born at <32 weeks GA (range: 24 + 0 to 31 + 6; mean: 28 + 3) and their clinical characteristics are summarized in Table 1.

### 3.2. EEG Data

EEG recording started at a median time of 7.7 (IQR: 5.0 to 16.5) hours after birth. Median duration of recording was 41.1 (IQR: 25.2 to 48.1) h. Three 1 h epochs per EEG were visually selected to be largely artifact-free, resulting in 87 epochs in total. The median PNA for the start of each 1 h EEG epoch was 26.9 h (IQR: 13.3 to 42.3 h).

### 3.3. EMA Estimations

**Figure 1:**
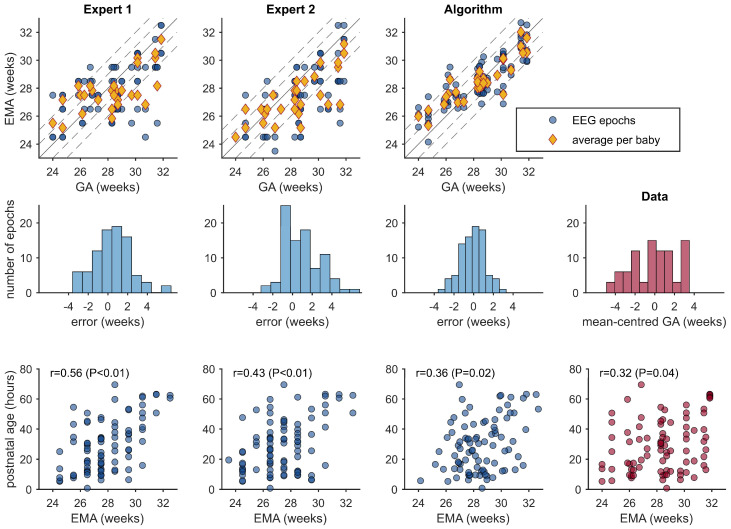
Accuracy of EEG maturational age (EMA) estimates of gestational age (GA) from 2 human experts and a computer algorithm. Estimates are made on 87, 1 h epochs (3 epochs per newborn). The average estimate per newborn is included in the top-row plots. Dashed lines in the top plots represent 1- and 2-week bounds. The middle-row plots show histograms of the EMA estimates minus GA, and the bottom row shows the association between the EMA estimate and postnatal age (PNA).

**Figure 2:**
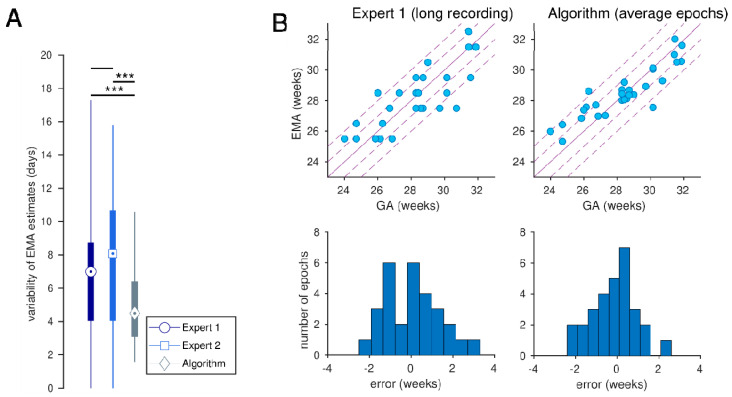
(**A**) Intra-rater analysis as the standard deviation of estimates over the 3 × 1 h EEG epochs (*** indicates *p* < 0.001); (**B**) EEG maturational age (EMA) estimates for expert 1, based on the long-duration recording and algorithm, averaging the estimates over the 3 epochs.

For the three × 1h epochs, the algorithm, comparative to the experts, has relatively higher correlation values and lower bias and SDE values (Figure 1 and Table 2). When averaging over the three epochs per newborn (Table 3), correlation was significantly higher and bias significantly lower (*p* < 0.05; see Table 4) for the algorithm compared to the experts. SDE was not significantly different (*p* = 0.175 and *p* = 0.200; Table 4). Between the experts’ EMA estimates, bias significantly differed (3.6 versus 7.0 days; *p* < 0.001) but correlation (*p* = 0.416) and SDE remained similar (*p* = 0.584). Intra-rater variability was significantly lower for the algorithm compared to the experts: SD between three epochs for the algorithm was 4.9 days and 7.4 days for expert 1 (*p* < 0.001) and 7.4 days for expert 2 (*p* < 0.001)—see Figure 2A. There was no difference in intra-rater variability between the experts (*p* = 0.954).

Estimating the EMA using the long-duration EEG trace for expert 1 improved performance—see Table 2. There was no significant difference in correlations or SDE between the human experts’ review of the long-duration EEG and the computer algorithm’s assessment of the average of the three × 1 h epochs: expert *r* = 0.816 versus algorithm *r* = 0.896 (*p* = 0.127); expert bias = 0.67 versus algorithm = −0.79 days (*p* = 0.335); expert SDE = 9.2 days versus algorithm SDE = 7.6 days (*p* = 0.243).

### 3.4. Mixed-Effects Models Postnatal Age

The best linear mixed-effects models for the two experts’ EMA estimation included both PNA and GA as fixed effects, without the interaction effect. These coefficients, detailed in Table 5, highlight the following: for expert 1, the EMA estimate is influenced by 6.6 days for a 1-day change in PNA (95% CI: 3.54 to 9.15 days); likewise for expert 2, the EMA estimate is influenced by up to 3.7 days for a 1-day change in PNA (95% CI: 0.47 to 6.90 days). R-adjusted values improve when PNA is included: 0.69 versus 0.59 for the expert 1 model and 0.68 versus 0.65 for the expert 2 model.

## 4. Discussion

In this study, we showed that an algorithm has similar or better performance compared to the visual estimation of EMA by experts for short-duration EEG recordings of preterm newborns with no major neurological abnormality at the time of the recording. We found a difference in the bias of estimates between the two experts but no difference in correlation or SDE values. The maturational features of preterm EEG are a keypoint in the assessment for prognostic purposes [13,15,16,31]. Therefore, establishing the reproducibility and reliability of the visual and automated interpretation of the EEG for estimating GA is crucial to highlight the role of EEG monitoring for assessing brain maturation and establishing prognosis.

In the present study, we aimed to focus on a selected population of preterm infants born at less than 32 weeks GA, monitored in the first 72 h from birth, without significant neurological impairment at the time of the EEG and favorable outcome, or adverse outcome due to later morbidity. Furthermore, we used long-duration, multi-channel EEG monitoring to analyze the possible effect of prolonged monitoring on the visual assessment of EMA. We also analyzed the effect of PNA on EMA estimation.

A previous study investigated the accuracy of, and agreement among, EEG readers’ estimation of maturity and a computer algorithm’s measure of EMA in preterm infants [25]. In this paper, short-duration EEG recordings up to the term equivalent age of infants born at <28 weeks GA were analyzed. Their findings align with ours and show that visual assessment of infant maturity is possible from the EEG or aEEG, with an average of human experts providing the highest accuracy with the algorithm providing the most accurate maturity assessment [25]. This study did not compare the accuracy of the human expert on longer-duration recordings, nor were the EEGs recorded within the first 72 postnatal hours. The authors also used a mixed population of both pathological and normal infants. While they did not show any effect of pathology on visual EMA assessment, we have eliminated any potential confounding effects by using infants with no serious neurological impairments at the time of recording.

In the present study, both experts analyzed short EEG recordings, knowing the recordings were within the first 72 h after birth but unaware of the exact PNA. A previous study has shown the influence of PNA on quantitative features of the preterm EEG by describing a clear, rapid trend of evolution over the first few days of age in excess of typical in-utero maturation [29]. The mixed-effects analysis presents a similar trend for visual EEG review: over the first 3 days of age, PNA can account for up to 12–20 days of the EMA estimate. It may also partially explain why the visual assessment of the full, long-duration EEG—with knowledge of PNA—produced an improvement over short-duration recording. The improvement of the human expert with the long-duration EEG may be also in relation to the higher amount of data available for visual assessment: a major possibility to check for specific age-dependent transients and a higher possibility to assess the features of the sleep–wake cycling. The EMA algorithm used in this paper includes PNA as a feature [29], and this may contribute to its increased performance over visual assessment of the 1h epochs. It is, therefore, advisable to include PNA in any preterm EEG assessment, particularly during the transitional period after birth.

### Limitations

The strict inclusion criteria used for this study meant that we could only include a small number of preterm newborns in the study. The selected GA range was also small, including only newborns <32 weeks GA. These limitations are also strengths of the study: we present a well-defined, homogeneous cohort of preterm newborns less than 32 weeks GA, recorded in the first 72 h of birth, without neurological complications at the time of the EEG recording, ensuring the assessment of an appropriate EEG activity for age. The algorithm has only been trained on this dataset, and the ability of the algorithm to generalize across a wide range of EEG recording environments should be tested with independent, held-out validation datasets. The scope for a wider gestational age range, up to term infants, is also untested. Unlike the visual interpretation of the human expert, these limitations are unique in the development of computer algorithms.

## 5. Conclusions

For short duration EEG recordings of 1h, the visual interpretation of the human expert tends to underestimate PMA. This results in a visual interpretation that is inferior to the accuracy of an age-prediction algorithm, thus making the algorithm a possibly useful tool in clinical practice in order to support the visual assessment of the EEG in preterm infants. Differences between the interpretation of the human expert and the algorithm are resolved when longer-duration recordings are reviewed. Quantitative EEG in preterms has been successfully applied to many applications of clinical relevance, such as tracking functional maturation, detecting, and even predicting early brain injury, and predicting long-term outcomes [23,28,29]. We believe that further developments of this approach would assist neurophysiologists and clinicians in assessing the EMA and, therefore, the trajectory of brain activity development in preterm infants.

## Figures and Tables

**Table 1:** Clinical characteristics, presented as either median (inter-quartile range) or sample size (percentage). Clinical risk for babies (CRIB) II, pH values, and Bayley scales III score evaluated for *n* = 26 infants.

Characteristic	*n* = 29
gestational age (weeks)	28.4 (26.7, 30.1)
female	21 (72.4%)
birth weight (g)	980 (800, 1240)
Apgar score at 1 m	8 (6, 8)
Apgar score at 5 m	9 (8, 9)
pH	7.21 (7.14, 7.28)
CRIB	8 (7, 10)
cesarean delivery	13 (44.8%)
Bayley scales III (26/29 newborns)	
cognitive	95 (95, 104)
language	103 (97, 111)
motor	100 (97, 106)

**Table 2:** Estimates of EEG maturational age (EMA) from human experts and the computer algorithm from 29 preterm newborns.

	Correlation	Bias (Days)	SDE (Days)	<1 Week (%)	<2 Weeks (%)
Mean GA in cohort		0.0 (−3.24, 3.37)	15.7 (13.8, 17.4)	31.0 (21.8, 41.4)	55.2 (44.8, 65.5)
Expert 1	0.597 (0.416, 0.737)	3.57 (0.83, 6.44)	13.2 (10.9, 15.3)	40.2 (29.9, 50.6)	72.4 (62.1, 81.6)
Expert 2	0.659 (0.530, 0.757)	7.03 (4.43, 9.61)	12.4 (10.6, 14.0)	46.0 (35.6, 56.3)	72.4 (62.1, 81.6)
Algorithm	0.833 (0.752, 0.890)	−0.79 (−2.75, 0.95)	8.7 (7.3, 10.0)	56.3 (44.8, 66.7)	88.5 (81.6, 94.3)
Expert 1 (long recording)	0.816 (0.671, 0.903)	0.67 (−2.64, 3.91)	9.2 (6.8, 11.3)	51.7 (34.5, 69.0)	86.2 (72.4, 96.6)
Algorithm	0.896	−0.79	7.6	69.0	93.1
(average epochs)	(0.794, 0.957)	(−3.80, 1.88)	(5.4, 9.4)	(51.7, 86.2)	(82.8, 100.0)

Performance of estimating the EEG maturational age (EMA) comparing the human experts’ interpretation of the long-duration EEG recording with the algorithm’s assessment averaged over the three × 1 h epochs. Metrics include Pearson’s correlation coefficient, bias and standard deviation of error (SDE) measured in days, and % of infants correctly classified within 7 days and within 14 days. The zero-mean distribution of the EMA data, as bias, SDE, and % within 1 and 2 weeks, is also included. Confidence intervals (95%), displayed in parentheses.

**Table 3:** Estimates of EEG maturational age (EMA) by averaging the estimates over the 3 epochs for each newborn.

	Correlation	Bias (Days)	SDE (Days)	<1 Week (%)	<2 Weeks (%)
Expert 1	0.704	3.57	11.3	44.8	75.9
	(0.438, 0.843)	(−0.35, 7.33)	(8.4, 13.5)	(27.6, 65.5)	(58.6, 89.7)
Expert 2	0.762	7.03	10.3	55.2	82.8
	(0.574, 0.902)	(3.42, 10.60)	(6.9, 12.8)	(37.9, 72.4)	(69.0, 96.6)
Algorithm	0.896	−0.79	7.6	69.0	93.1
	(0.799, 0.958)	(−3.56, 2.00)	(5.4, 9.4)	(51.7, 82.8)	(82.8, 100.0)

Metrics include Pearson’s correlation coefficient, bias, and standard deviation of error (SDE) measured in days, and % of infants correctly classified within 7 days and within 14 days. Confidence intervals (95%), displayed in parentheses.

**Table 4:** Statistical comparison of the correlation, bias, and standard deviation of error (SDE) between the human experts and the computer algorithm estimates of EEG maturational age (EMA). To account for repeated measures per newborn, metrics from Table 2 are reassessed by averaging the estimates over the 3 epochs per newborn (*n =* 29 for each test; metrics in Table 3). Pairwise *p*-values are corrected for multiple comparisons using the Holm procedure; unadjusted values are in parentheses.

	Correlation	Bias	SDE
	*p* a	*p* b	*p* c
Expert 1 vs. Expert 2	0.416 (0.416)	0.009 (0.009)	0.584 (0.584)
Expert 1 vs. EMA	0.006 (0.002)	0.006 (0.003)	0.175 (0.059)
Expert 2 vs. EMA	0.049 (0.024)	<0.001 (<0.001)	0.200 (0.399)

^a^ William’s test for dependent correlations; ^b^ Paired *t*-test; ^c^ Brown–Forsythe test.

**Table 5:** Fixed-effects estimates from a linear mixed-effects model using EEG maturational age (EMA) as the predictor variable. Confidence intervals at 95% are in parentheses. Key: GA, gestational age; PNA, postnatal age.

	Expert 1		Expert 2	
	Coefficient	*p*-Value	Coefficient	*p*-Value
intercept (days)	106.4 (77.7, 136.2)	<0.001	81.4 (45.1, 117.5)	<0.001
PNA (days)	6.57 (3.54 to 9.15)	<0.001	3.74 (0.47, 6.90)	0.021
GA (days)	0.407 (0.251, 0.554)	<0.001	0.533 (0.349, 0.724)	<0.001

## Data Availability

The original contributions presented in this study are included in the article. Further inquiries can be directed to the corresponding author.

## Abbreviations

The following abbreviations are used in this manuscript:

EMA	EEG maturational age
PNA	Post-natal age
GA	Gestational age
NICU	Neonatal Intensive Care Unit
ICH-GCP	International Conference on Harmonisation of Technical Requirements for Registration of Pharmaceuticals for Human Use of Good Clinical Practice
IVH	Intra-ventricular hemorrhage
PVL	Periventricular leukomalacia
EEG	Electroencephalogram
SD	Standard deviation
SDE	Standard deviation error
CRIB	Clinical risk for babies
IQR	Interquartile range
